# The Application of Chitosan for Protection of Cultural Heritage Objects of the 15–16th Centuries in the State Tretyakov Gallery

**DOI:** 10.3390/ma15217773

**Published:** 2022-11-04

**Authors:** Alexander Zhgun, Darya Avdanina, Balzhima Shagdarova, Gulgina Nuraeva, Kirill Shumikhin, Yuliya Zhuikova, Alla Il’ina, Egor Troyan, Michail Shitov, Valery Varlamov

**Affiliations:** 1 Research Center of Biotechnology, Russian Academy of Sciences, 119071 Moscow, Russia; 2 State Tretyakov Gallery, 119017 Moscow, Russia

**Keywords:** chitosan, biodeterioration of cultural heritage, antiseptic, benzalkonium chloride, sodium pentachlorophenolate, Fourier transform infrared spectroscopy, attenuated total reflection, atomic force microscopy

## Abstract

Microorganisms are one of the main factors in the deterioration of cultural heritage, in particular art paintings. The antiseptics currently used in painting have significant limitations due to insufficient effectiveness or increased toxicity and interaction with art materials. In this regard, the actual challenge is the search for novel materials that effectively work against microorganisms in the composition with painting materials and do not change their properties. Chitosan has pronounced antimicrobial properties but was not used previously as an antiseptic for paintings. In our study we developed a number of mock layers based on sturgeon glue, supplemented which chitosan (molecular weight 25 kDa or 45 kDa), standard antiseptics for paintings (positive controls) or without additives (negative control). According to Fourier transform infrared spectroscopy and atomic force microscopy, the addition of chitosan did not significantly affect the optical and surface properties of this material. The ability of chitosan to effectively protect paintings was shown after inoculation on the created mock-up layers of 10 fungi-destructors of tempera painting, previously isolated from cultural heritage of the of the 15–16th centuries in the State Tretyakov Gallery, on the created mock layers. Our study demonstrated the principled opportunity of using chitosan in the composition of painting materials to prevent biodeterioration for the first time.

## 1. Introduction

For their growth, microorganisms are capable of efficiently utilizing many organic materials from which cultural heritage objects are created [1,2,3,4,5]. The primary rule for preserving cultural objects from biodegradation in the conditions of museum storage is strict observance of conservation conditions, including the temperature and humidity regimes, which do not allow the growth of microorganisms [6]. However, these measures are not always sufficient due to various factors, such as: (i) local deviations from standard storage conditions or (ii) the appearance in the microbiome of the museum of microorganisms adapted to extreme conditions of existence, for example, xerophilic fungi that survive in increased dryness [6,7,8]. In this regard, antimicrobial biocides are used as an important additional measure in conservation and restoration practice [9,10,11,12]. There are a huge number of antiseptic substances, such as alcohols, aldehydes, phenols, acids, acid esters, amides, carbamates, dibenzamidines, pyridines, azoles, heterocycles, activated halogen compounds, surface active agents, organometallics, and oxidizing agents [6,13,14], but only a few of them are used for the conservation and restoration of cultural heritage. The number of biocidal products suitable for the treatment of cultural heritage is significantly limited, as only a small number of agents have been tested for their compatibility with historical materials such as pigments and organic binders, and there are very few studies on the long-term effects of biocides such as possible color changes or decomposition products [6,15,16]. The latest approaches associated with biocleaning are currently under development and have not been introduced into routine restoration practice [17,18,19,20]. One of the main substances used in world practice in this regard are: (i) compounds that release formaldehyde [21,22]; (ii) isothiazolinone, which has an effective and preventive effect for the treatment of paper carriers [23]; as well as (iii) quaternary ammonium salts with carbon chain length C8–C18, for example, benzalkonium chloride (BAC) [24,25]. The number of compounds that are used as antiseptics for the protection of paintings has recently been significantly reduced due to the abandonment of previously used highly toxic substances [26,27,28,29]. The use of such a narrow range of compounds significantly limits the possibilities of antiseptic treatment of paintings. In particular, systematic treatment with a widely-used BAC selects organisms resistant to this compound [25]. We have previously shown that even the most active antiseptics, such as sodium pentachlorophenolate (NaPCP), do not completely protect painting materials from biodeterioration [30]. In this regard, there is an active screening for novel materials that could be used as antiseptics for paintings [31]. Thus, searching for new non-toxic and broad-spectrum antiseptics that do not interact and lose their properties in the composition of paintwork materials is an urgent task [30].

Chitosan is a natural biopolymer with a wide and varied range of applications [32,33,34]. One of the branches of application is related to the antimicrobial activity of chitosan [35,36,37]. Numerous studies have shown the activity of chitosan against both bacteria [38,39] and fungi [40,41]. It was also shown that various modifications can enhance the biocidal activity of chitosan [42,43]. However, until the 2020s, the possibility of using chitosan as an antiseptic to protect cultural heritage, including paintings, from biodegradation was not studied. Recently, scientists have made a successful attempt to use chitosan-based nanoparticles as a carrier for thymol to protect a cultural heritage site (Feilaifeng limestone, Hangzhou, China) against *Aspergillus niger* [44]. In 2020, we made the first attempt to study the possibility of using chitosan to protect cultural heritage paintings from microbial damage [45]. To do this, we studied the activity of low molecular weight (LMW) chitosan against 10 moldy fungi isolated from tempera paintings of the 15–16th centuries in the State Tretyakov Gallery (STG, Lavrushensky pereulok, 10, Moscow, Russia) and capable of biodegrading paintwork materials [30,45]. In particular, fungi were isolated from icon “the Church Militant” (dated 1550s), bust fragment of the statue “Holy Great Martyr George the Victorious” (1464, Lime Stone, tempera) and icon “Holy Great Martyr Demetrius of Thessaloniki” (dated 16th century). These molds have been extensively studied both as test cultures for screening the activity of novel alkyl nucleosides [46,47,48] and for potential applications in biotechnology [49]. The drop and dilution screening on Czapek–Dox agar (CDA) medium preliminarily revealed the antifungal properties of all tested chitosans, with molecular weights (MW) of 6, 12, 18, 25, and 45 kDa [45]. Moreover, chitosans with MW of 25 and 45 kDa had the strongest inhibitory effect against studied test cultures from STG.

However, many substances may change their chemical and/or physical properties when added to the composition of materials [50]. Thus, antiseptics that are active on standard microbiological media may lose their activity in composition with painting materials or change their physicochemical properties in an undesirable way. In this regard, the aim of our work was to study the possibility of using LMW chitosan (with MW 25 and 45 kDa) as an antiseptic in the composition of sturgeon glue (used to bond painting materials to canvas). 

To do this, at the first stage of our work, we quantified the concentrations of chitosans that inhibit the metabolic activity of ten strains of filamentous fungi isolated from cultural heritage objects in the State Tretyakov Gallery [30]. It was important to carry out such experiments in order to select the necessary concentrations of chitosans for the preparation of mock layers. We then created a series of mock layers (models) based on sturgeon glue with the addition of LMW chitosan (or control antiseptics used in the restoration of paintings) and studied their spectral, surface, and bioprotective properties against dominant fungi-destructors of tempera paintings from the microbiome of STG. The obtained results make it possible to consider chitosan as a promising antiseptic for painting materials, which has a number of advantages compared to the substances used. 

## 2. Materials and Methods

### 2.1. Materials

Low molecular weight chitosan was obtained by chemical depolymerization of high molecular weight chitosan, molecular weight (MW) 500 kDa, degree of deacetylation (DD) 85%, with 6 M HCl, 90 °C, 3 h, as described previously [51]. Chitosan samples MW 25 kDa and 45 kDa and DD 97–99%, polydispersity index 1.6–1.85 were obtained. Degree of deacetylation of chitosans was determined by ^1^H NMR method. ^1^H NMR spectra of chitosan were recorded on a Bruker AMX 400 spectrometer (Billerica, MA, USA) operating at 1H frequency of 400 MHz at 32 °C.

Iodonitrotetrazolium chloride (INT), 1-methoxy-phenazine methosulfate (1-methoxy-PMS), and dimethyl sulfoxide (DMSO) were from Merck, Germany. Benzalkonium chloride (BAC, commercial name Katamin AB) was from Neochemax, (Domodedovo, Russia); sodium pentachlorophenolate (NaPCP) from IndiaMART (Noida, Uttar Pradesh, India). Wooden boards were from LLC Mytishchi Woodworking Plant (Mytishchi, Moscow region, Russia); pavoloka (canvas) was from LLC Belarusian Len-Ivanovo (Ivanovo, Russia); chalk was from JSC Shebekinsky Chalk Plant (Shebekino, Russia). Sturgeon glue was from LLC Condor (Moscow, Russia). 

### 2.2. Obtaining Mock Layers (Models or Mock-Ups) Based on Sturgeon Glue with Additions of Antiseptics

On birch boards with a size of 23.5 × 14.5 cm, a canvas (pavoloka) was pasted, pre-treated with a 10% aqueous solution of sturgeon glue. Materials dried for 1 day at room temperature, after this 4 layers of levkas (ground layer, 8% aqueous solution of sturgeon glue-sifted chalk, 1:3, *v*/*v*) were applied. Then the surface was leveled with sandpaper, the workpieces were fragmented on a circular machine into separate fragments to create blanks, which were then used to obtain various mock layers.

For this, a 7% aqueous solution of sturgeon glue was prepared at 55–60 °C in a water bath, to which 1% chitosan with MM 25 kDa, or 1% chitosan with 45 kDa, or 1% BCA, or 1% NaPCP were added, or nothing was added (negative control). These 5 mixtures, based on 7% aqueous solution of sturgeon glue, were applied in 3 layers to the surface of blanks to obtain mock layers (models, or mock-ups), types **I**–**V** (Table 1). Then, a small triangular fragment of ~10 mm^2^ was cut from one corner for analysis by Fourier transform infrared spectroscopy (FTIR) and atomic force microscopy (AFM). The rest of the mock-up material was used to determine the bioprotective properties of developed materials against fungal test cultures. Each layout type was made in 6 replicates, 3 of which were then named variant **a** (**Ia**, **IIa**, **IIIa**, **IVa**, and **Va**) and used to inoculate group **A** strains (*Aspergillus versicolor* STG-25G, *Simplicillium lamellicola* STG-96, *Aspergillus creber* STG-57, *Cladosporium halotolerans* STG-52B, and *Aspergillus versicolor* STG-86), while the other 3 mock-ups were named variant **b** (**Ib**, **IIb**, **IIIb**, **IVb**, and **Vb**) and were used to inoculate group **B** strains (*Microascus paisii* STG-103, *Aspergillus creber* STG-93W, *Cladosporium parahalotolerans* STG-93B, *Aspergillus protuberus* STG-106, and *Ulocladium* sp. AAZ-2020a STG-36). Thus, variants **a** and **b** of the same mock layer type were identical in material composition (Table 1). 

### 2.3. Fourier Transform Infrared Spectroscopy (FTIR) of Selected Materials and Mock Layers

Infrared (IR) spectra of chitosan powders (MW 25 kDa and 45 kDa), flakes of sturgeon glue, and created mock layers No. **I**–**V** (Section 2.2) were acquired using Nicolet™ iS50 FTIR Spectrometer (Thermo Fisher Scientific, Waltham, MA, USA) in the wavenumber range from 600 to 4000 cm^−1^ (64 scans at the 4.0 cm^−1^ resolution). Attenuated total reflection (ATR) method with diamond crystal was used. The probed area was 120 × 120 μm^2^. Spectra were processed and analyzed using the OMNIC software (Thermo Fisher Scientific, Waltham, MA, USA). For all the resulting spectra a spectral window of 2400–2250 cm^−1^ corresponding to the CO_2_ bands from the atmosphere was flattened. The spectra of mock layers No. **I** and No. **II** were compared with the spectra of the initial chitosan powders with MW 25 kDa and 45 kDa, respectively. To determine the contribution of individual materials in a mock layer mixture and increase the resolution of the overlapping bands, the 2nd derivatives from spectra of mock layers No. **I** and No. **II** were obtained using the OMNIC program. Then the main bands of 25 kDa and 45 kDa chitosan spectra (1066, 1033, 1531, 1381, and 1417 cm^–1^) were compared with the bands of the obtained spectra of the 2nd derivatives of No. **I** and No. **II** in the OMNIC software, respectively.

### 2.4. Atomic Force Microscopy (AFM) of Mock Layers 

The surface of created mock layers **I**–**V** (Section 2.2.) was characterized using an Integra Prima atomic force microscope NT-MDT SI (LLC “NT-MDT”, Moscow, Russia) in air. The scanning process was carried out in a semi-contact mode with a frequency of 1.1–1.4 Hz, with a resolution of 512 × 512 pixels. Silicon cantilevers of the Etalon HA_FM series (TipsNano, Tallinn, Estonia) with a cantilever resonant frequency of 114 kHz, a probe curvature radius of less than 10 nm, and a force constant of about 6.0 N/m were used. The scan results were processed using NOVA and Image Analysis P9 software (NT-MDT SI, Moscow, Russia). Statistical surface parameters (ASME B46.1) such as root mean square (RMS) roughness of the surface (Sq), maximum area peak height (Sp), and maximum area valley depth (Sv) were determined by analyzing five images (for each of the sizes: 2 × 2, 5 × 5, 10 × 10, and 20 × 20 µm) for each sample from mock layers. The mean values and standard deviations of the mean were calculated. 

### 2.5. Microorganism Strains Used in the Work

Ten micromycete strains, isolated previously from the exhibits and surfaces of the “Halls of Painting of Ancient Rus” (No. 56, 57 and 61) in the main historical building of the State Tretyakov Gallery (10 Lavrushinsky per., Moscow, Russia) [30], were used as test cultures to determine the antimycotic activity of studied compounds. *Aspergillus versicolor* STG-25G (SRX7729174; MK260015.1) and *Ulocladium* sp. AAZ-2020a STG-36 (MW590700.1; SRX7729176) were isolated from the icon “the Church Militant” (dated 1550s). *Cladosporium halotolerans* STG-52B (SRX7729178; MK258720.1) was isolated from a bust fragment of the statue “Holy Great Martyr George the Victorious” (1464, Lime Stone, tempera). *Aspergillus creber* STG-57 (SRX7729151; MK266993.1) was isolated from the icon “Holy Great Martyr Demetrius of Thessaloniki” (dated 16th century). *Aspergillus versicolor* STG-86 (SRX7729182; MK262781.1), *Aspergillus creber* STG-93W (SRX7729186; MW575292.1), *Cladosporium parahalotolerans* STG-93B (SRX7729188; MK262909.1), *Simplicillium lamellicola* STG-96 (SRX7729192; MK262921.1) were isolated from the surfaces of hall №61. *Microascus paisii* STG-103 (SRX7729190; MW591474.1) was isolated from the hall №57. *Aspergillus protuberus* STG-106 (SRX7729192; MK268342.1) was isolated from the hall №56. 

### 2.6. Cultivation of Fungal Strains on Standard Microbiological Media

The filamentous fungi, isolated from STG, were cultivated on slant agarized Czapek–Dox (CDA) medium (30 g/L sucrose, 2 g/L NaNO_3_, 1 g/L K_2_HPO_4_, 0.5 g/L MgSO_4_ × 7 H_2_O, 0.5 g/L KCl, 0.01 g/L FeSO_4_ × 7 H_2_O, 20 g/L agar, pH 7.0–7.4) at 24–26 °C.

### 2.7. Measuring the Effect of Chitosans on the Metabolic Activity (MA) of STG Strains

The effect of chitosans (with MW 25 kDa and 45 kDa) on metabolic activity (MA) of ten fungal test cultures from STG was analyzed using a modified tetrazolium method (MTT test) as described previously, with some modification [52]. After 7–10 days of cultivation fungal cells were collected from CDA slant agar with 10 mL of potato dextrose broth (PDB, 4 g/L (from 200 g infused potato), 20 g/L glucose, pH 5.4–5.8) and filtered through sterile cotton to remove myceleum. The conidia contained in the supernatant were diluted in PDB to a stock concentration of 2.5 × 10^5^ conidia/mL. Chitosan powders with MW 25 kDa or 45 kDa were preliminarily dissolved in 0.5% acetic acid to a stock concentration of 20 mg/mL, diluted in the first well in PDB to 10 mg/mL, and then serial two-fold dilutions were made in PDB to 0.08 mg/mL. The corresponding serial two-fold dilutions of acetic acid were prepared for the control. Assay to determine the MA was performed in flat-bottomed 96-well plates (SPL Life Sciences, Pocheon-si, Korea). For this, 100 µL of the stock solution of conidia was added to 100 µL of serially two-fold diluted chitosans, to a final range of 0.04–5 mg/mL (or to controls with acetic acid). Samples in plates were incubated for 24 h at 25 °C. Then, 10 μL of solution A (5 mg/mL INT (dissolved in 0.1 M sodium phosphate buffer, pH 7.4), 1-methoxy-PMS 4 mg/mL) was added and incubated for 4 h at 37 °C. The supernatant was carefully decanted; 150 µL of 100% DMSO was added to the colored precipitate to dissolve the resulting formazan crystals. Dissolution was carried out for 3 h at 37 °C with mixing at 100 rpm. The absorbance was measured at 540 nm with a microplate photometer (Thermo Scientific Multiskan FC, USA). The fungal MA was calculated by the formula: MA = OD_t_/OD_c_ × 100%, where OD_t_ indicates the absorbance in treatment set, and OD_c_ indicates the absorbance in control set. To assess the MA of the studied fungi, the EC_50_ value was used, corresponding to 50% inhibition of fungal metabolism. The data recorded were measured in triplicate and repeated at least three times.

### 2.8. Determination of the Antiseptic Properties of Chitosan in Mock Layers 

To determine the antiseptic properties of chitosan in mock layers, the drop dilution method was used as previously described [53,54] with some modifications. After 10–15 days of cultivation fungal cells were collected from CDA slant agar, diluted with 0.9% NaCl to 4 × 10^6^ CFU/mL (designated as dilution 10^–1^), and sequential tenfold dilutions were performed in 0.9% NaCl to 4 × 10^5^ and 4 × 10^4^ CFU/mL (designated as dilutions 10^–2^ and 10^–3^, respectively). Before inoculation, mock layers in sterile Petri dishes were preliminarily saturated with 0.3 mL H_2_O/1 cm^3^ at 25 °C, 48 h. In order to avoid direct contact of the material with water, mock layers were placed on sterile hydrophobic pads. Then, drops of 2.5 μL from dilutions 10^−1^–10^−3^ (which correspond to 10^5^, 10^4^, and 10^3^ CFU/drop) were inoculated onto pre-saturated mock-up layers **I**–**V**, then incubated at 25 °C, 42 days. A certain five test cultures were co-inoculated on variant **a** of each mock layer (**Ia**–**Va**), and the rest five test cultures were co-inoculated on variant **b** of each mock layer (**Ib**–**Vb**), Table 2. The inhibitory effects of the compounds were measured every seven days after inoculation. To determine the percent of fungal growth inhibition (FGI) used the formula: FGI% = [(Dc − Dt)/Dc] × 100, where Dc indicates the colony diameter in control set, and Dt indicates the colony diameter in treatment set. The data recorded were measured in triplicate and repeated at least twice.

## 3. Results

### 3.1. Effect of Chitosans on the Metabolic Activity (MA) of STG Strains

**Figure 1:**
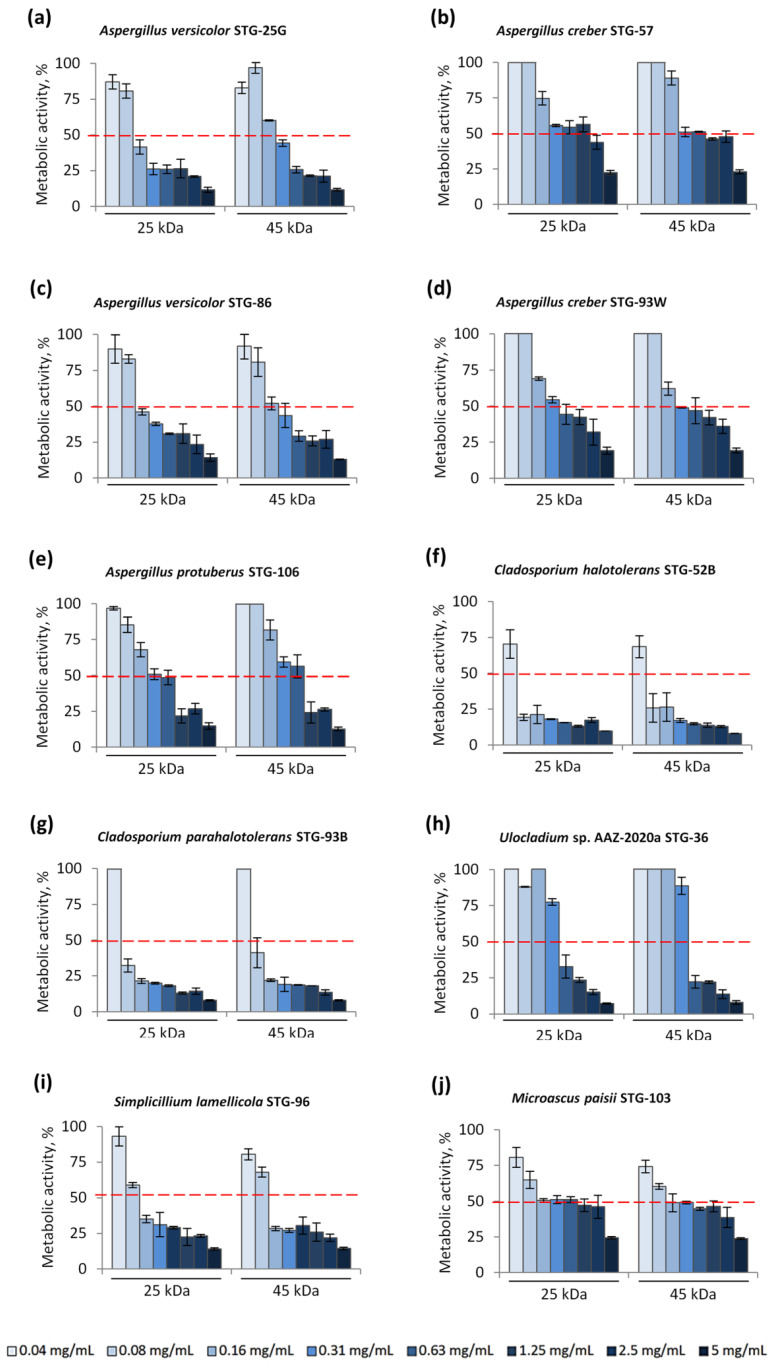
Influence of low molecular weight chitosans on the metabolic activity (MA) of fungi-destructors of painting materials, isolated in the State Tretyakov Gallery: 25 kDa—chitosan with MW 25 kDa; 45 kDa—chitosan with MW 45 kDa. The red dotted line shows the level corresponding to the EC_50_ (50% inhibition of MA). Data are presented as mean ± SD, *n* = 3.

The effect of chitosans on the metabolic activity of test cultures was quantified as a percentage of the MA of the strain on a potato dextrose broth (PDB) with chitosan compared with the growth of fungi on the control medium by the modified tetrazolium method (MTT test). As test cultures, 10 representatives of filamentous fungi, which are known to have biodeterioration activity against organic materials used in tempera painting of the 15–16th centuries, were used [30]. Five species of fungi were from the family *Aspergillaceae* (*Aspergillus versicolor* STG-25G, *A. creber* STG-57, *A. versicolor* STG-86, *A. creber* STG-93W, *A. protuberus* STG-106), two species were from the family *Cladosporiacea* (*Cladosporium halotolerans* STG-52B, *C. parahalotolerans* STG-93B) and one representative each from the families *Pleosporaceae* (*Ulocladium* sp. AAZ-2020a STG-36), *Cordycipitaceae* (*Simplicilium lamellicola* STG-96) and *Microascaceae* (*Microascus paisii* STG-103). These strains were dominant among the entire microbiome that could be found in STG and were documented by metagenomic profile characterization [30]. Assays were carried out for chitosans with a MW of 25 kDa and 45 kDa, which showed the highest activity in previous experiments on fungal growth inhibition (FGI) with a fixed concentration of chitosans in CDA medium [45]. Inhibition of fungal metabolism was assessed using the concept of EC_50_, which corresponds to 50% inhibition of MA (Figure 1).

It turned out that members of the *Cladosporiaceae* family are the most sensitive to the action of chitosan (Figure 1f,g), which correlates with our previous data [45]. MA of strains *C. halotolerans* STG-52B and *C. parahalotolerans* STG-93B varied in the range of 7–25% at chitosan concentrations ≥0.08 mg/mL. Representatives of the families *Pleosporaceae* (*Ulocladium* sp. AAZ-2020a STG-36) and *Cordycipitaceae* (*S. lamellicola* STG-96) under the influence of chitosans showed EC_50_ at concentrations ≥0.63 mg/mL and ≥0.16 mg/mL, respectively (Figure 1h,i). Members of the *Aspergillaceae* family were more resistant to chitosan. EC_50_ for strains *A. versicolor* STG-25G and *A. versicolor* STG-86 was at chitosan concentrations ≥0.31 mg/mL (Figure 1a,c), for *A. creber* STG-93W was at chitosan concentrations ≥0.63 mg/mL (Figure 1d), for *A. protuberus* STG-106 (Figure 1e) was at chitosan concentrations ≥1.25 mg/mL, and for *A. creber* STG-57 was in the concentration range of 2.5–5 mg/mL (Figure 1b). Very close resistance was demonstrated by the representative of the family *Microascaceae*; EC_50_ for the strain *M. paisii* STG-103 was in the concentration range of 2.5–5 mg/mL (Figure 1j). *Aspergillaceae* showed increased resistance to chitosan compared to other strains, which is consistent with our previous results on agar medium [45]. At the same time, the strain *M. paisii* STG-103, which is relatively sensitive to chitosan on CDA medium, showed additional resistance in the current experiment on PDB medium. This may be due to the fact that the CDA medium is not optimal for this test culture, on which *M. paisii* STG-103 exhibits the slowest radial mycelium growth among STG strains.

The MW of the studied chitosans did not significantly affect the inhibition of fungal MA (Figure 1). When diluted, chitosans with MW of 25 kDa and 45 kDa were added to the cells of fungal strains, very close trends in inhibitory of MA were observed. At the same time, as in previous experiments on CDA medium [45], some differential effects on *Aspergillus* were found. For example, *A. versicolor* STG-25G and *A. versicolor* STG-86 strains were more sensitive to chitosan with MW of 25 kDa: EC_50_ (25 kDa) = 0.16 mg/mL, EC_50_ (45 kDa) = 0.31 mg/mL (Figure 1a,c); while the *A. creber* STG-93W strain, on the contrary, was more sensitive to chitosan with MW of 45 kDa: EC_50_ (25 kDa) = 0.63 mg/mL, EC_50_ (45 kDa) = 0.31 mg/mL (Figure 1d). Both types of LMW chitosans were used for further study. 

### 3.2. Obtaining Mock Layers with the Addition of LMW Chitosans or Control Antiseptics

**Figure 2:**
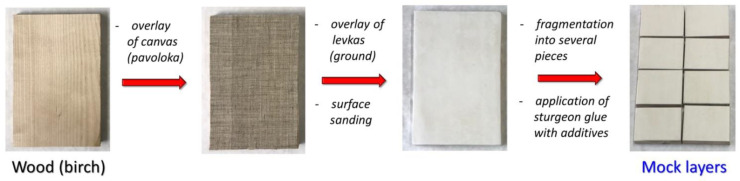
The main stages of obtaining mock layers based on sturgeon glue with additions of low MW chitosans and control antiseptics.

In the current study, we created a series of mock layers (models or mock-ups) to determine the antifungal activity of chitosan in the composition of painting materials, according to a previously described procedure [30]. The main stages of obtaining mock layers, including (i) the imposition of a canvas (pavoloka) on a wooden base; (ii) applying of levkas (ground) and polishing the surface; (iii) fragmentation into several pieces; and (iv) application of sturgeon glue with additives are shown in Figure 2. 

As additives to sturgeon glue we used chitosans with MW of 25 and 45 kDa, which showed the best antifungal characteristics in previous experiments [45], and which we further characterized in the current study (Section 3.1). To create positive control mock-ups, we used additives of standard antiseptics used in painting and scientific restoration, such as BAC and NaPCP. Based on previous results on FGI by LWW chitosan in CDA [45] and current data on the inhibition of MA by LMW chitosan in PDB, we chose a 1% concentration to create mock layers with chitosans. We used the same 1% concentration for the addition of control standard antiseptics (BAC and NaPCP). We also made mock layers without any additives for negative control.

### 3.3. Analysis of the Physical Properties of Mock Layers

#### 3.3.1. FTIR Analysis of Initial Raw Materials and Developed Mock Layers

**Figure 3:**
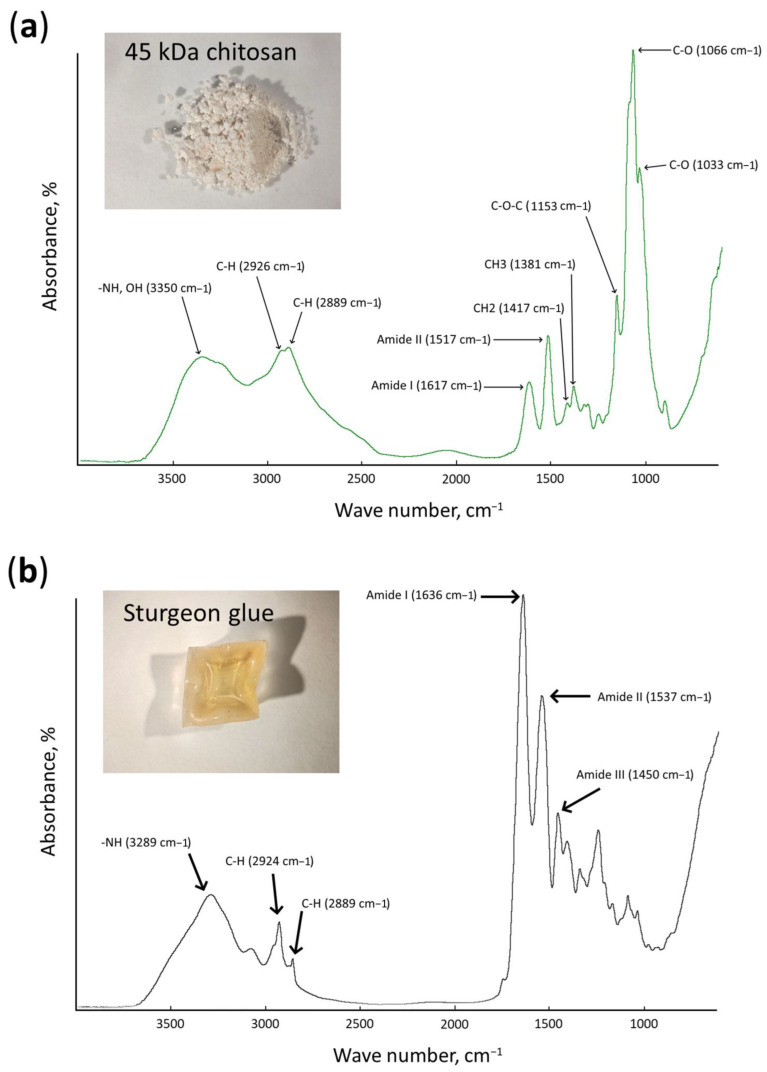
FTIR spectra of initial raw materials: (**a**)—chitosan with MW 45 kDa; (**b**)—sturgeon glue.

**Figure 4:**
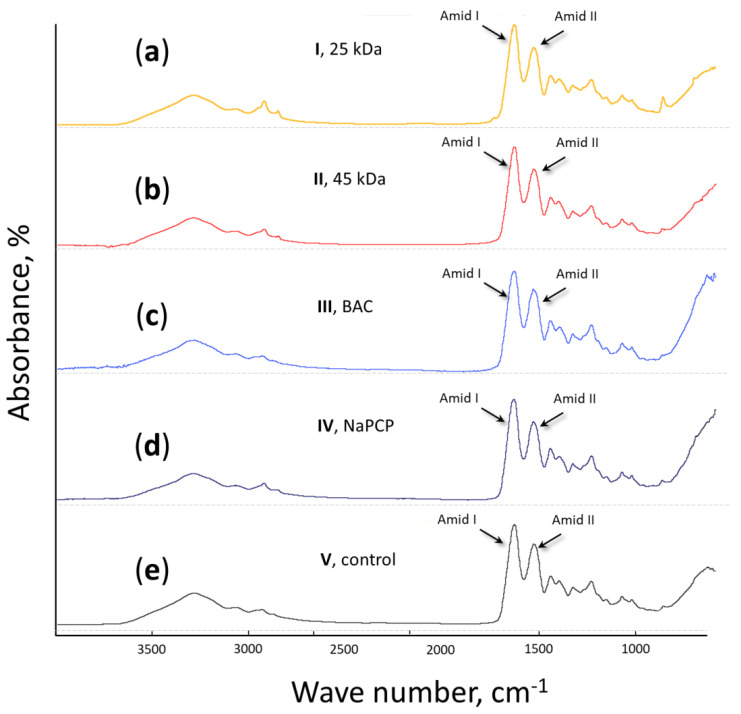
FTIR spectra of mock layers No. **I**–No. **V**. Sturgeon glue additives: (**a**)—chitosan with MW 25 kDA; (**b**)—chitosan with MW 45 kDA; (**c**)—benzalkonium chloride (BAC); (**d**)—sodium pentachlorophenolate (NaPCP); (**e**)—without additives (control). All additives were in the amount of 1%.

To characterize the effect of chitosan additives on the spectral characteristics of the obtained mock-up layers, we performed an FTIR analysis of initial raw materials, sturgeon glue and LMW chitosans, and composite materials, mock layers No. **I**–No. **V** (Figure 3, Figure 4, and Figures S1 and S2). It turned out that the FTIR spectra of all mock layers (No. **I**–No. **V**) were very close to the original spectrum of sturgeon glue (Figure 3b and Figure 4). The addition of 1% standard antiseptics such as BAC or NaPCP (to mocks layers No. **III** and No. **IV**, respectively) did not make a significant difference compared to the control mock layer No. **V** (Figure 4c–e). Additionally, the introduction of 1% LMW chitosans into the composition of the mock-up material does not have a detectable effect on FTIR spectra compared to the control (Figure 4a,b,e).

For an accurate quantitative determination of the contribution of 1% chitosans (with a MW of 25 kDa and 45 kDa) to the spectral properties of a mock layer based on sturgeon glue, the spectra of initial powders of chitosans with MW 25 kDA and 45 kDA were compared with modified spectra of mock-ups No. **I** and No. **II**, respectively (Figures S1 and S2). The resolution of spectra No. **I,** and No. **II** were increased by transformation back into an interferogram, then multiplied by a function the nth derivative. For further analysis, the 2nd derivative was used, for which the best resulting spectra were obtained. The resulting spectra of mock layers had sharper bands than the original, which helped to increase the resolution of the overlapping bands. The aim was to see if minor additions of chitosan could be detected using FTIR spectroscopy. Then the main bands of chitosan spectra (1066, 1033, 1531, 1381, and 1417 cm^–1^) were compared with the bands of the obtained spectra of the 2nd derivative of the mock layers’ spectra. Amide bands of chitosan were not taken into account due to the presence of strong amide bands of sturgeon glue (Figure 3b). The comparison showed that at a given concentration (1%), chitosan demonstrated little to no contribution in the resulting spectra and so cannot be traced with IR spectroscopy as part of the layout material.

Thus, it was shown that in models based on sturgeon glue in the mid-IR range used for the qualitative and quantitative analysis of organic compounds, the addition of 1% NM chitosan was not observed.

#### 3.3.2. AFM Analysis of the Developed Mock Layers

**Figure 5:**
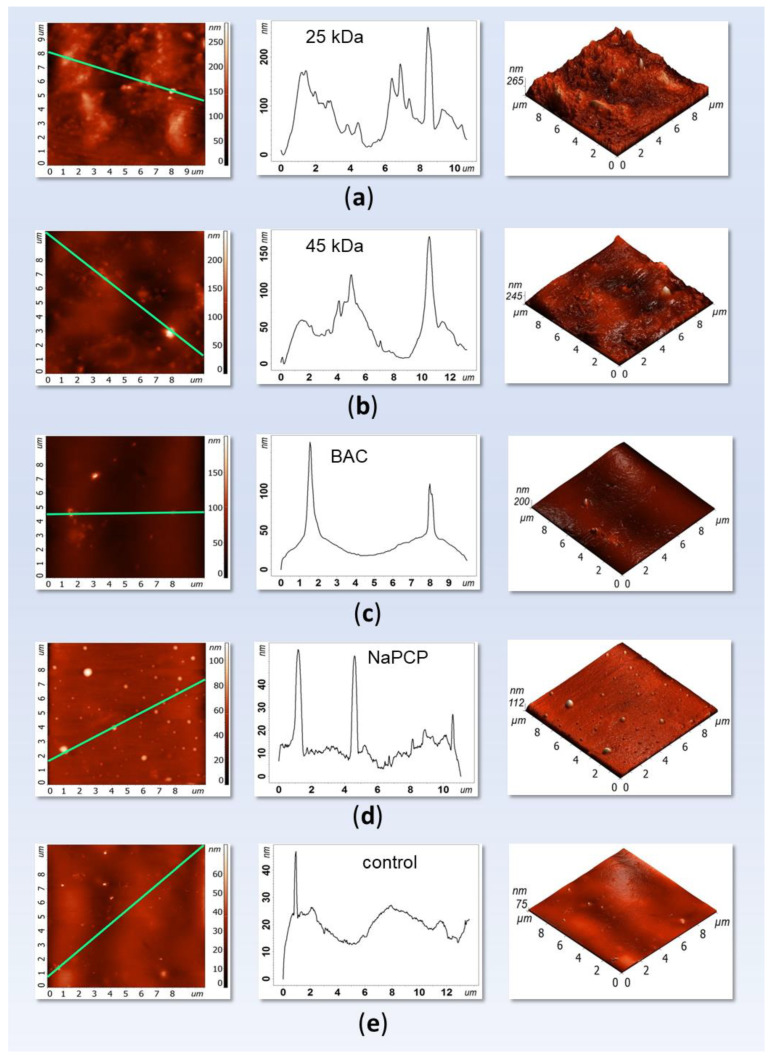
AFM images of mock layers No. **I**–No. **V**. Scanning area—10 × 10 µm. Sturgeon glue additives: (**a**)—chitosan with MW 25 kDa; (**b**)—chitosan with MW 45 kDa; (**c**)—benzalkonium chloride (BAC); (**d**)—sodium pentachlorophenolate (NaPCP); (**e**)—without additives (control). All additives were in the amount of 1%.

In order to determine the effect of the addition of LMW chitosans and control antiseptics on surface roughness, the obtaining mockups were analyzed by the AFM method (Figure 5 and Figure S3). It turned out that the average RMS roughness of the surface, parameter Sq, in LMW chitosans (mock layers **I**, **II**) is 2–2.5 times higher than in the control (mock layer **V**), Table 3. The addition of BAC does not affect the average RMS roughness of the surface of the material (mock layer **III**); the addition of NaPCP leads to a significant decrease in average Sq, approximately three times (mock layer **IV**). Along with this, an important indicator is the spread at the parameter Sq. For the control sample, it is more than 80% (±9.5, with an average value of Sq = 11.6). The addition of LMW chitosans, along with an increase in the average value of Sq, leads to a decrease in the spread of this parameter to 30% (25 kDa chitosan) and to 10% (45 kDa chitosan). Also, the addition of BAC reduces the spread of the mean Sq to 20%, and the addition of NaPCP reduces the spread of the mean Sq to 45%.

AFM analysis showed that the addition of all analyzed substances makes the surface of sturgeon glue more homogeneous, which is expressed by a significant decrease in the spread of the Sq parameter. At the same time, RMS roughness of the surface of materials based on chitosan lies in the upper scatter area Sq of the control, RMS roughness of the surface with the addition of NaPCP lies in the lower scatter area Sq of the control, and RMS roughness of the surface with the addition of BAC lies at the level Sq of the control with less spread. It is known that animal glues can have an undesirable tendency to foam, developing small air bubbles in the glue matrix which can disrupt the uniformity of the dried glue film and weaken bonds [55]. When characterized by atomic force microscopy, this can be expressed in a large scatter of the Sq parameter (Table 3). Possibly, LMW chitosans play the role of high scaffolds, making the material more homogeneous, removing undesirable foaming; since the chitosan molecules themselves have a characteristic size, when they fill the cavities of the bubbles, the value of parameter Sq increases.

Thus, it has been shown that the addition of the studied LMW chitosan does not have a significant effect on the surface of the created models, which is important for the use of painting materials [56]. 

### 3.4. Analysis of the Antiseptic Properties of LMW Chitosan in Mock Layers

Since at the previous stage of the work we showed that the addition of chitosan does not have a significant effect on the spectral and surface properties of the material (Section 3.3), we were able to proceed to the next and main stage of our work, the determination of the antifungal properties of low molecular weight chitosan in the composition of painting materials. The antifungal activity of chitosan with MW of 25 kDa and 45 kDa, as well as BAC and NaPCP as a positive control was determined in the composition of the created mock layers based on sturgeon glue (Figure 1, Table 1). All compounds were added in an amount of 1%. As test cultures, we used the same mold strains from STG that we previously worked with on standard microbiological media, such as Czapek–Dox agar medium [45] or liquid media (Section 3.1).

**Figure 6:**
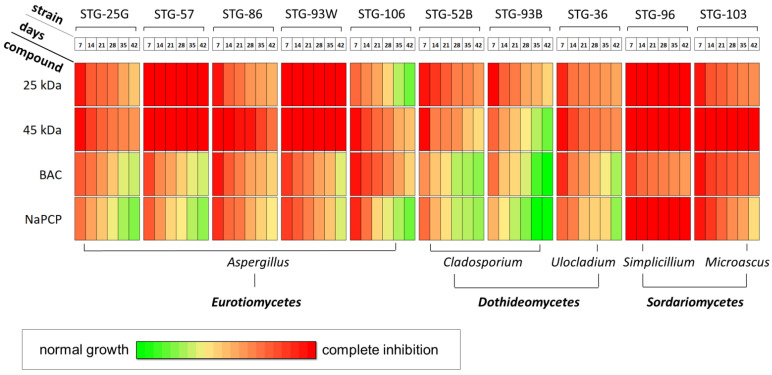
The dynamics of growth inhibition (%) for test cultures on mock-up layers with the addition of selected antifungal compounds: 25 kDa—chitosan with MW 25 kDa; 45 kDa—chitosan with MW 45 kDa; BAC—benzalkonium chloride; NaPCP—sodium pentachlorophenolate. Data on 7, 14, 21, 28, 35, and 42 days after inoculation.

Fungal growth inhibition (FGI) data were obtained every 7 days after inoculation of test cultures on mock-ups for 42 days (Figure 6, Figures S4 and S5).

**Figure 7:**
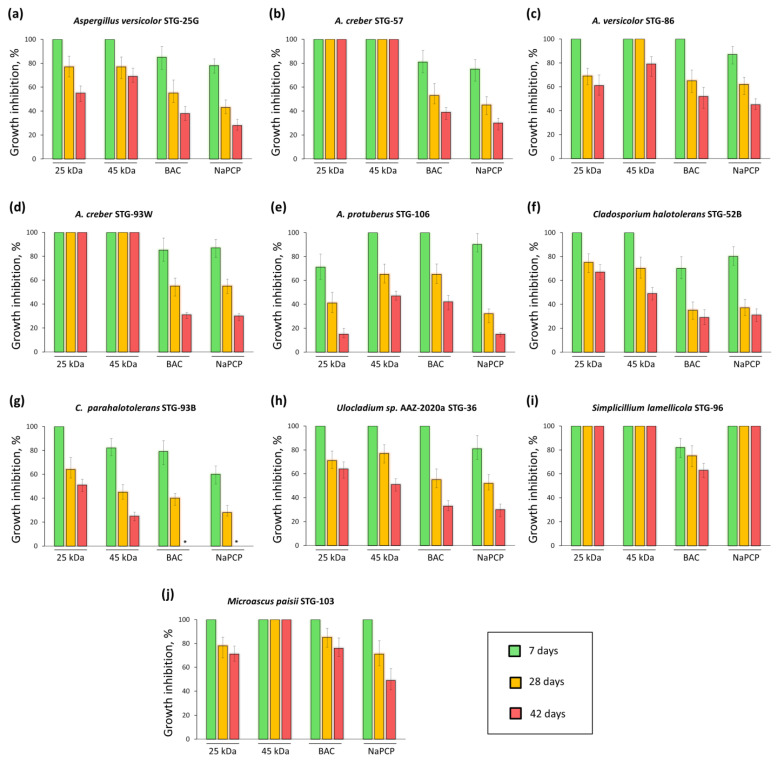
Growth inhibition (%) of STG strains on mock-up layers with the addition of selected antifungal compounds: 25 kDa—chitosan with MW 25 kDa; 45 kDa—chitosan with MW 45 kDa; BAC—benzalkonium chloride; NaPCP—sodium pentachlorophenolate. Data acquired at 7, 28, and 42 days after inoculation. Data are presented as mean ± SD, *n* = 3. The symbol * means “not detected”.

Seven days after inoculation, 100% FGI was observed on mock-ups with the addition of both types of chitosan for most test cultures, with the exception of FGI_[25kDa,7d]_ = 71% for *A. protuberus* STG-106 and FGI_[45kDa,7d]_ = 82% for *C. parahalotolerans* STG-93B (Figure 7e,g). At the same time, standard antiseptics in general showed somewhat worse results when assessing the growth of 7-day inoculums. Among *Aspergillus*, BAC inhibited 100% only *A. versicolor* STG-86 and *A. protuberus* STG-106 (Figure 7c,e). For strains *A. versicolor* STG-25G, *A. creber* STG-57, and *A. creber* STG-93W FGI_[BAC,7d]_ = 81–85% (Figure 7a,b,d). With the addition of NaPCP 100% inhibition was not observed for any of the *Aspergillus*, for these five strains FGI_[NaPCP,7d]_ was =75–90% (Figure 7a–e). For *Cladosporium* strains (*C. halotolerans* STG-52B and *C. parahalotolerans* STG-93B), inhibition by standard antiseptics was less than for *Aspergillus*: FGI_[BAC,7d]_ = 70–79%, FGI_[NaPCP, 7d]_ = 60–80% (Figure 7f,g). BAC and NaPCP completely inhibited the growth of *M. paisii* STG-103 (Figure 7j) and had different effects on *Ulocladium* sp. AAZ-2020a STG-36 (for which FGI_[BAC,7d]_ = 100%, FGI_[NaPCP,7d]_ = 81%; Figure 7h) and *S. lamellicola* STG-96 (for which FGI_[BAC,7d]_ = 82%, FGI_[NaPCP,7d]_ = 100%; Figure 7i).

With further cultivation, for most strains, the appearance or increase in radial growth was observed on models with antiseptics, which was quantitatively expressed in a decrease in FGI (Figure 6 and Figure 7). Thus, 28 days after *Aspergillus* inoculation on mock layers with chitosan (mock layers I and II) 100% inhibition was observed in five out of ten cases: for *A. creber* STG-57 (FGI_[25kDa,28d]_ = 100% and FGI_[45kDa,28d]_ = 100%), *A. versicolor* STG-86 (FGI_[45kDa,28d]_ = 100%), and *A. creber* STG-93W (FGI_[25kDa,28d]_ = 100% and FGI_[45kDa,28d]_ = 100%). In the remaining five cases, FGI decreased by 21–35% compared with FGI after 7 days. So for *A. versicolor* STG-25G: FGI_[25kDa,28d]_ = 77% and FGI_[45kDa,28d]_ = 77%; for *A. versicolor* STG-86: FGI_[45kDa,28d]_ = 79%; for *A. protuberus* STG-106: FGI_[25kDa,28d]_ = 41% and FGI_[45kDa,28d]_ = 65%. *Cladosporium* generally followed the same trend as *Aspergillus*, FGI decreased by 25–37% compared with FGI after 7 days. So for *C. halotolerans* STG-52B: FGI_[25kDa,28d]_ = 75% and FGI_[45kDa,28d]_ = 70%; for *C. parahalotolerans* STG-93B: FGI_[25kDa,28d]_ = 64% and FGI_[45kDa,28d]_ = 45%. The addition of both chitosans completely inhibited the growth of *S. lamellicola* STG-96; only 45 kDa completely inhibited the growth *M. paisii* STG-103 (FGI_[25kDa,28d]_ = 78%, FGI_[45kDa,28d]_ = 100%); for *Ulocladium* sp. AAZ-2020a STG-36 FGI decreased by 23–29% compared with FGI after 7 days (FGI_[25kDa,28d]_ = 71%, and FGI_[45kDa,28d]_ = 77%). For cultures inoculated on mock layers, supplemented with standard antiseptics, after 28 days, a similar trend was generally observed, as for cultures inoculated on mock layers supplemented with chitosans, FGI values decreased (Figure 7). However, such decrease was generally stronger, compared to day 7, reaching 43% for the value differences between FGI_[NaPCP,7d]_ and FGI_[NaPCP,28d]_ for strain *C. halotolerans* STG-52B (Figure 7f). The absolute values of FGI, i.e., for all 28 days of cultivation, for mock-ups with added chitosans were also somewhat better. This is also indicated by minimum FGI values of specific antiseptics against strains most resistant to them: 25 kDa chitosan vs. *A. protuberus* STG-106 (FGI_[25kDa,28d]_ = 41%), 45 kDa chitosan vs. *C. parahalotolerans* STG-93B (FGI_[45kDa,28d]_ = 45%), BAC vs. *C. halotolerans* STG-52B (FGI_[BAC,28d]_ = 35%), and NaPCP vs. *A. protuberus* STG-106 (FGI_[NaPCP,28d]_ = 32%).

By the final stage of experiment, after 42 days of cultivation, both LMW chitosan completely inhibited the growth of *A. creber* STG-57, *A. creber* STG-93W and *S. lamellicola* STG-96. In addition, 45 kDa completely inhibited the growth of *M. paisii* STG-103. Among standard antiseptics, 100% GFI was observed in only one case, for NaPCP vs. *S. lamellicola* STG-96. This strain was found to be the most sensitive to the effects of antiseptics, the addition of three of the four compounds caused complete growth suppression, the addition of BAC inhibited growth by about a third (FGI_[BAC,42d]_ = 63%). Another representative of *Sordariomycetes*, strain *M. paisii* STG-103, in addition to increased sensitivity to 45 kDa (FGI_[45kDa,42d]_ = 100%), demonstrated: FGI_[25kDa,42d]_ = 71%, FGI_[BAC,42d]_ = 76%, FGI_[NaPCP,42d]_ = 49%. In general, representatives of the *Sordariomycetes* class of fungi turned out to be the most sensitive to the studied compounds (Figure 6). On the other hand, representatives of the *Dothideomycetes* class of fungi demonstrated increased resistance; for example, for the *Cladosporium* family there were such values as: *C. halotolerans* STG-52B (FGI_[25kDa, 42d]_ = 67%, FGI_[45kDa,42d]_ = 49%, FGI_[BAC,42d]_ = 29%, FGI_[NaPCP,42d]_ = 31%); *C. parahalotolerans* STG-93B (FGI_[25kDa,42d]_ = 51%, FGI_[45kDa,42d]_ = 25%, FGI_[BAC,42d]_ = 0%, FGI_[NaPCP,42d]_ = 0%). That is, standard antiseptics ceased to have an inhibitory effect against *C. parahalotolerans* STG-93B after 42 days, while the efficiency of chitosan remained at the level of 25–51%. Another representative of *Dothideomycetes* (from the *Ulocladium* family) also showed increased resistance 42 days after inoculation, and this resistance was higher with respect to standard antiseptics than to chitosan: *Ulocladium* sp. AAZ-2020a STG-36 (FGI_[25kDa,42d]_ = 64%, FGI_[45kDa,42d]_ = 51%, FGI_[BAC,42d]_ = 33%, FGI_[NaPCP,42d]_ = 30%).

Representatives of the class *Eurotiomycetes* (genus *Aspergillus*) occupied an intermediate position in terms of tested compounds between *Sordariomycetes* and *Dothideomycetes*, showing relatively moderate resistance (Figure 6). At the same time, some representatives of *Aspergillus* showed both increased resistance (*A. protuberus* STG-106) and increased sensitivity (*A. creber* STG-57, *A. versicolor* STG-86). With regard to *A. protuberus* STG-106, chitosans showed rather low values: FGI_[25kDa,42d]_ = 15%, FGI_[45kDa,42d]_ = 47%. This correlates with our previous data on the CDA medium showing that *A. protuberus* STG-106 exhibits the highest resistance to LMW chitosan among the studied STG strains [45].

**Figure 8:**
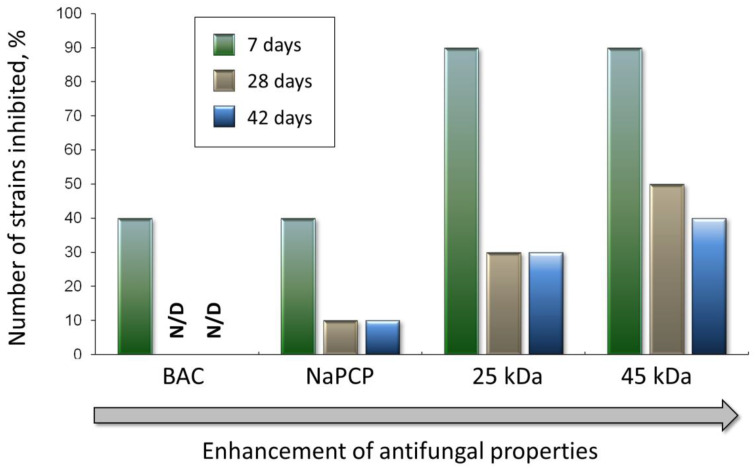
The percentage of completely inhibited fungal STG microbiome (among the 10 studied strains) at 7, 28, and 42 days after inoculation on mock-up layers with the addition of selected antifungal compounds: 25 kDa—chitosan with MW 25 kDa; 45 kDa—chitosan with MW 45 kDa; BAC—benzalkonium chloride; NaPCP—sodium pentachlorophenolate. N/D—not detected.

To compare the antiseptic effect of chitosan and standard antiseptics, we summarized the data on the dynamics of growth inhibition of test cultures on mock-up layers (Figure 6 and Figure 7), determining the percentage of completely inhibited fungal STG microbiome at 7, 28, and 42 days after inoculation (Figure 8).

On the 7th day after inoculation, standard antiseptics completely inhibited the growth (FGI = 100%) of 40% of strains, and chitosans completely inhibited the growth of 90% of strains (Figure 8). After the 28th day after inoculation on mock layers with the addition of BAC, all strains showed growth. On mock layers, supplemented with NaPCP, only 10% of STG-strains were completely inhibited; this value did not change at the end of the experiment, after 42 days. The percentage of completely inhibited strains also significantly decreased during cultivating on mock layers with the addition of chitosans. In particular, on mock layer No. I (chitosan with MW 25 kDa), after 28 days of cultivation, growth was completely inhibited in 30% of strains, this value did not change until the end of the experiment; on mock layer No. II (chitosan with MW 45 kDa) after 28 days, the number of completely inhibited strains decreased to 50%, by day 42 it decreased further to 40%.

## 4. Discussion

Previously, we worked with mock-ups based on sturgeon glue, with the addition of 1% NaPCP [30]. At this concentration, the growth of microorganisms was significantly reduced compared to the control (mock layers based on sturgeon glue without the addition of an antiseptic), but it was not completely suppressed. Therefore, at such a sublethal concentration, it was possible to quantitatively compare the strength of the antiseptic. Additionally, in our previous work, we found that LMW chitosan exhibited antifungal sublethal doses in CDA media over a concentration range of 0.5–2% [45]. In the current study we additionally specified the effective concentrations for the most active chitosans, determined the EC_50_ for STG strains (Figure 1). It turned out that for most test cultures EC50 ≤ 0.125% (1.25 mg/mL), and only for two strains EC50 is in the range of 0.25–0.5% (2.5–5 mg/mL) (Figure 1b,j). It is significant that in the liquid nutrient medium we saw the main trends that we previously described for these fungal strains in CD agar medium: *Cladosporium* were the most sensitive, and *Aspergillus* were resistant to chitosan exposure (Figure 1, [45]). At the same time, the studied strains were generally more sensitive to chitosan in a liquid nutrient medium than on the agar medium. Based on these data, it was decided to use a 1% concentration of chitosan to prepare mock-ups, which presumably could effectively protect the material and not significantly affect its properties. To determine the required sensitivity of test cultures, corresponding to their sublethal level, three doses of cells of each strain, diluted at a concentration of 10^3^, 10^4^, and 10^5^ CFU/drop, were used for inoculation. Experiments have shown that 1% concentration of all tested compounds (LMW chitosan, BAC, and NaPCP) turned out to be sublethal for test cultures inoculated at 10^5^ CFU/drop, which made it possible to quantitatively compare antiseptic properties in mock layers (Figure 6, Figure 7 and Figure 8).

After choosing the concentrations of chitosans and developing mock layers with their addition (Figure 2), it was necessary to characterize possible changes in the materials. After we selected the concentrations of chitosans and made mock layers with them (Figure 2), it was necessary to characterize possible changes in the materials. Antiseptics used in painting should not affect the spectral characteristics of painting materials [30]. This key requirement is aimed at preserving the color authenticity of the painting [57]. FTIR is one of the main methods for analyzing painting materials [58]. Typical absorption patterns in the IR spectrum make it possible to identify substances present in the sample [59]. In our work, we demonstrated by the power of FTIR analysis that the addition of 1% LMW chitosan does not affect the spectral characteristics of mock layers based on sturgeon glue. To do this, we determined the FTIR spectra of LMW chitosan and sturgeon glue, the material to which chitosan was added (Figure 3, Figures S1 and S2). We also determined the FTIR spectra for mock-ups created with and without the addition of chitosan (Figure 4). It turned out that the main peaks of the FTIR spectrum for sturgeon glue coincide with the peaks of the created mock-ups (Figure 3 and Figure 4). In addition, the FTIR spectra for mock layers No. **I** and No. **II** do not show peaks corresponding to the added 1% chitosan with a MW of 25 kDa (Supplementary Materials, Figure S1) or 45 kDa (Supplementary Materials, Figure S2). In previous work, we have shown that the addition of 1% NaPCP does not affect the FTIR spectrum of sturgeon-based mockups [30]. In the current study, we demonstrated that another standard antiseptic for painting materials, BAC, also does not affect the FTIR spectrum of mock-ups based on sturgeon glue (Figure 4). In our experiments, LMW chitosans, like standard antiseptics, did not contribute to the resulting spectrum at the working concentration. This may indicate that chitosan can also be used to protect painting materials. Final conclusions can be drawn after experiments on artificial aging of mock layers with the addition of chitosan and the study of their spectral characteristics.

All test cultures used in the work belong to the so-called moldy fungi. A moldy fungi (also called molds or *Filamentous fungi*) is a taxonomically diverse organisms from phylum Zygomycota and Ascomycota with filamentous hyphae and has the ability to produce airborne spores or conidia [60]. Some of these fungi are extensively studied as they are also important sources for the production of pharmaceutically significant drugs such as antibiotics, statins, and immunosuppressants [61,62,63,64]. However, only recently moldy fungi began to be actively studied at the molecular genetic level in relation to the ability of their individual representatives to destroy cultural heritage sites [9,30,65]. The appearance of such works opens up opportunities for the development of new antiseptics aimed against the microbiome of a particular museum [46,47], for example, based on LMW chitosans [45].

As can be seen from the results obtained, all the studied substances exhibit antiseptic properties in the composition of the created layouts (Figure 6 and Figure 7). In general, representatives of the *Sordariomycetes* class of fungi (genera: *Simplicillium* and *Microascus*) turned out to be the most sensitive to the studied compounds, *Dothideomycetes* (genera: *Cladosporium* and *Ulocladium*) turned out to be the most resistant, and *Eurotiomycetes* (genus *Aspergillus*), showed relatively moderate resistance (Figure 6). At the same time, LMW chitosans (with MW 25 kDa and 45 kDa) generally work more efficiently than standard antiseptics used in restorative practice, such as BAC and NaPCP (Figure 6, Figure 7 and Figure 8). Moreover, if we take the percentage of strains in which the antiseptic completely inhibits growth as a criterion for evaluation, then chitosan with MW 45 kDa has slightly better antiseptic properties than chitosan with MW 25 kDa (Figure 8).

It is important that chitosans showed complete inhibition of both strains of *A. creber* (STG-57, STG-93W), since *A. creber* belongs to the so-called xerophilic fungi that can live at low humidity (up to 65–70% and below) [7]. It is rather difficult to shield cultural heritage from xerophilous fungi, since they can grow even if the temperature and humidity regime and other museum conservation rules are strictly observed [7]. It is noteworthy that standard antiseptics failed to completely inhibit these strains (STG-57, STG-93W) starting from the 7th day after inoculation on test mock-ups (Figure 7b,d). It is possible that resistant strains from the microbiome were selected against these traditionally used antiseptics, as shown for BAC in the antiseptic treatment at Lascaux Cave, a cultural heritage site [25]. In this regard, previously unused compounds based on LMW chitosan may be considered as promising antiseptics.

Since the studied chitosan in compositions with sturgeon glue proved capable of effectively protecting the tested materials from dominant mold fungi, it seems possible to use chitosan-based antiseptics as targeted agents against fungi-destructors of paintwork materials at the State Tretyakov Gallery. In addition, further studies with microorganisms from other museums may show how wide the spectrum of action of LMW chitosan against microorganisms that destroy paintings.

## 5. Conclusions

The current study shows for the first time that low molecular weight chitosan can exhibit effective antifungal properties in the composition of the painting material sturgeon glue. The concentrations used (1%), effective against mold fungi, do not have a significant effect on the spectral and surface properties of sturgeon glue. This result is important because animal glues used in painting to bond canvas and paint material are highly susceptible to biological attack. At the same time, the number of effective antiseptics used in painting is extremely limited. The data obtained in this work open up the possibility of further developments in the field of studying chitosan to protect cultural heritage objects from biodegradation.

## Figures and Tables

**Table 1:** Materials used to develop the mock layers.

No.	Materials for Mock Layer Development		Purpose
	Additive to Sturgeon Glue, %	Other Materials	
**I**	25 kDa chitosan, 1%	Wood, canvas, levkas	tested material
**II**	45 kDa chitosan, 1%		
**III**	BAC, 1%		positive control
**IV**	NaPCP, 1%		
**V**	–		negative control

**Table 2:** The order of inoculation test cultures on mock layers.

No.	Inoculum
**Ia–Va**	*Aspergillus versicolor* STG-25G
	*Simplicillium lamellicola* STG-96
	*Aspergillus creber* STG-57
	*Cladosporium halotolerans* STG-52B
	*Aspergillus versicolor* STG-86
**Ib–Vb**	*Microascus paisii* STG-103
	*Aspergillus creber* STG-93W
	*Cladosporium parahalotolerans* STG-93B
	*Aspergillus protuberus* STG-106
	*Ulocladium* sp. AAZ-2020a STG-36

**Table 3:** Parameters of the surface roughness of analyzed mock layers ^1^.

Mock Layer	Additive	Parameter ^2^	Mean Value, nm
**I**		Sq	28.5 ± 8.6
	25 kDa chitosan	Sp	167.2 ± 23.3
		Sv	116.3 ± 13.0
**II**		Sq	24.7 ± 5.6
	45 kDa chitosan	Sp	158.2 ± 59.5
		Sv	96.2 ± 43.4
**III**		Sq	11.8 ± 2.6
	BAC	Sp	163.4 ± 64.9
		Sv	40.6 ± 8.2
**IV**		Sq	3.8 ± 1.7
	NaPCP	Sp	40.4 ± 15.5
		Sv	29.4 ± 7.3
**V**		Sq	11.6 ± 9.5
	–	Sp	91.5 ± 64.9
		Sv	40.0 ± 15.1

^1^ Size of scan area was 10 × 10 µm. ^2^ Sq—root mean square roughness of the surface; Sp—maximum area peak height, Sv—maximum area valley depth.

## Data Availability

The data presented in this study are contained within the article.

## Supplementary Materials

The following supporting information can be downloaded at: https://www.mdpi.com/article/10.3390/ma15217773/s1, Figure S1: Determination of the contribution of 1% chitosan (with a MW of 25 kDa) to the spectral properties of a mock layer No. **I** (based on sturgeon glue, supplemented with 25 kDa chitosan). FTIR spectra of: (a)—chitosan with MW 25 kDa; (b)—mock layer No. **I**; (c)—superposition of (a) and (b). (d)—2nd derivative of (b); Figure S2: Determination of the contribution of 1% chitosan (with a MW of 45 kDa) to the spectral properties of a mock layer No. **II** (based on sturgeon glue, supplemented with 45 kDa chitosan). FTIR spectra of: (a)—chitosan with MW 45 kDa; (b)—mock layer No. **II**; (c)—superposition of (a) and (b). (d)—2nd derivative of (b); Figure S3: AFM images of mock layers No. **I**—No. **V**. Scanning area (from left to right)—2 × 2, 5 × 5, and 20 × 20 µm. Sturgeon glue additives: (a)—chitosan with MW 25 kDa; (b)—chitosan with MW 45 kDa; (c)—benzalkonium chloride (BAC); (d)—sodium pentachlorophenolate (NaPCP); (e)—without additives (control). All additives were in the amount of 1%; Figure S4: Inoculation of fungal test cultures on mock layer No. **V**a. Inoculums: A—*Aspergillus versicolor* STG-25G, B—*Simplicillium lamellicola* STG-96, C—*Aspergillus creber* STG-57, D—*Cladosporium halotolerans* STG-52B, and E—*A. versicolor* STG-86. 2.5 µL of 10-fold diluted inoculums were added: 10^−1^, 10^−2^ and 10^−3^, corresponds to 10^5^, 10^4^, and 10^3^ CFU. The upper right corner of the layout is cut off for the orientation of the application of inoculants; Figure S5: Growth of test cultures on mock-ups layers based on sturgeon glue with the addition of selected antifungal compounds (from left to right): chitosan with MW 25 kDa, chitosan with MW 45 kDa, BAC—benzalkonium chloride, NaPCP—sodium pentachlorophenolate. 14 days after inoculation, 25 °C. Drops of 2.5 μL from dilutions 10^−1^–10^−3^ of fungal cells (which correspond to 10^5^, 10^4^, and 10^3^ CFU/drop) were used for inoculation of strains: *Aspergillus versicolor* STG-25G, *Simplicillium lamellicola* STG-96, *A. creber* STG-57, *Cladosporium halotolerans* STG-52B, *A. versicolor* STG-86, *Microascus paisii* STG-103, *A. creber* STG-93W, *C. parahalotolerans* STG-93B, *A. protuberus* STG-106, and *Ulocladium* sp. AAZ-2020a STG-36.

## Abbreviations

AFM	Atomic force microscopy
ATR	Attenuated total reflection
BAC	Benzalkonium chloride
CDA	Czapek–Dox agar
DD	Degree of deacetylation
DMSO	Dimethyl sulfoxide
FGI	Fungal growth inhibition
FTIR	Fourier transform infrared spectroscopy
INT	Iodonitrotetrazolium chloride
IR	Infrared
LMW	Low molecular weight
MA	Metabolic activity
MW	Molecular weight
NaPCP	Sodium pentachlorophenolate
PDB	Potato dextrose broth
PMS	Phenazine methosulfate
RMS	Root mean square
STG	State Tretyakov Gallery (museum, Moscow, Russia)
