# Supplementary figures and images for: The Application of Chitosan for Protection of Cultural Heritage Objects of the 15–16th Centuries in the State Tretyakov Gallery
Source: Materials (Basel). 2022 Nov 4;15(21):7773. doi: 10.3390/ma15217773 (PMC9658413; doi:10.3390/ma15217773)

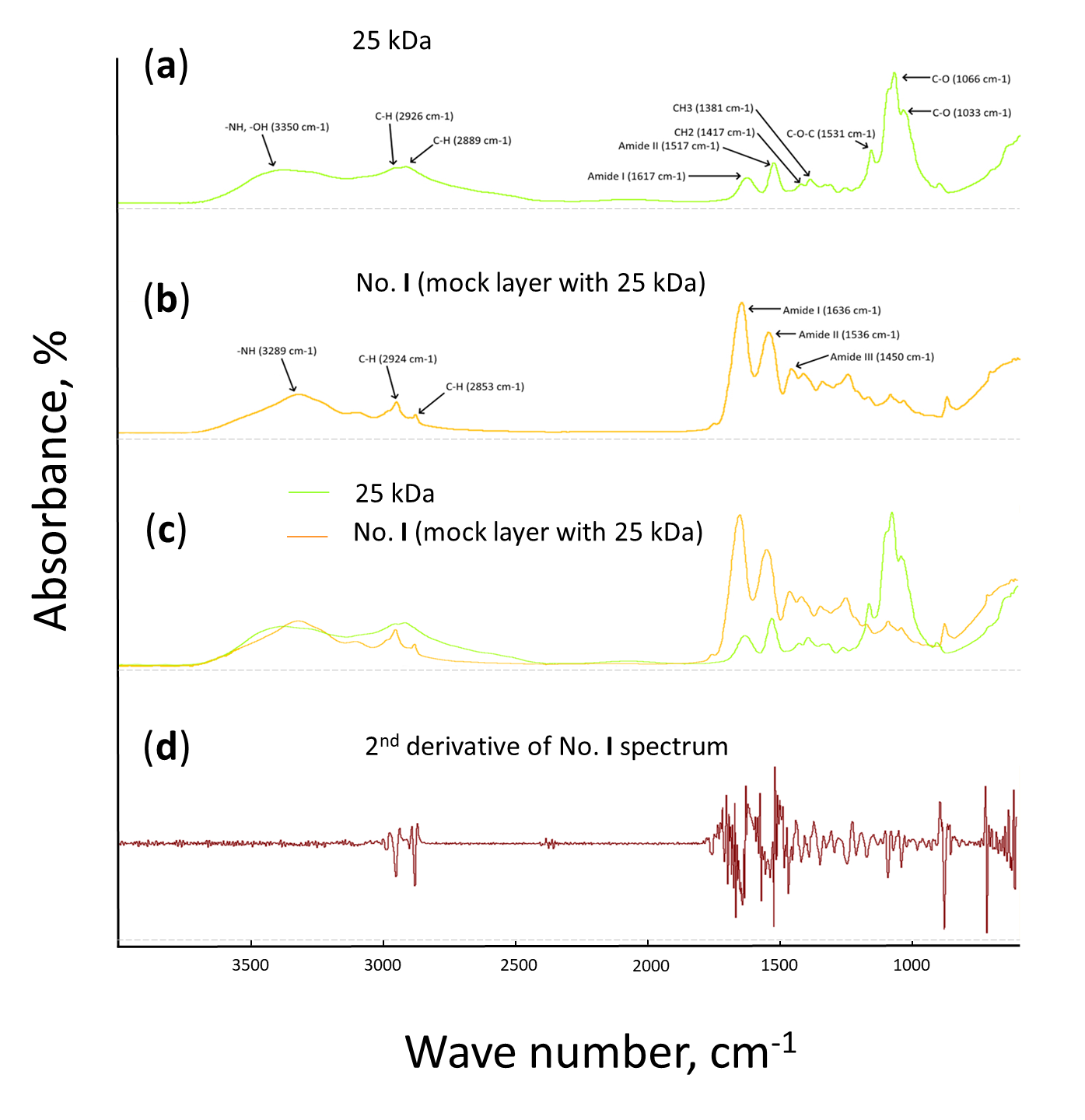

Supplement: Supplementary file 1 [file materials-15-07773-s001.zip › Figure S1.tif]

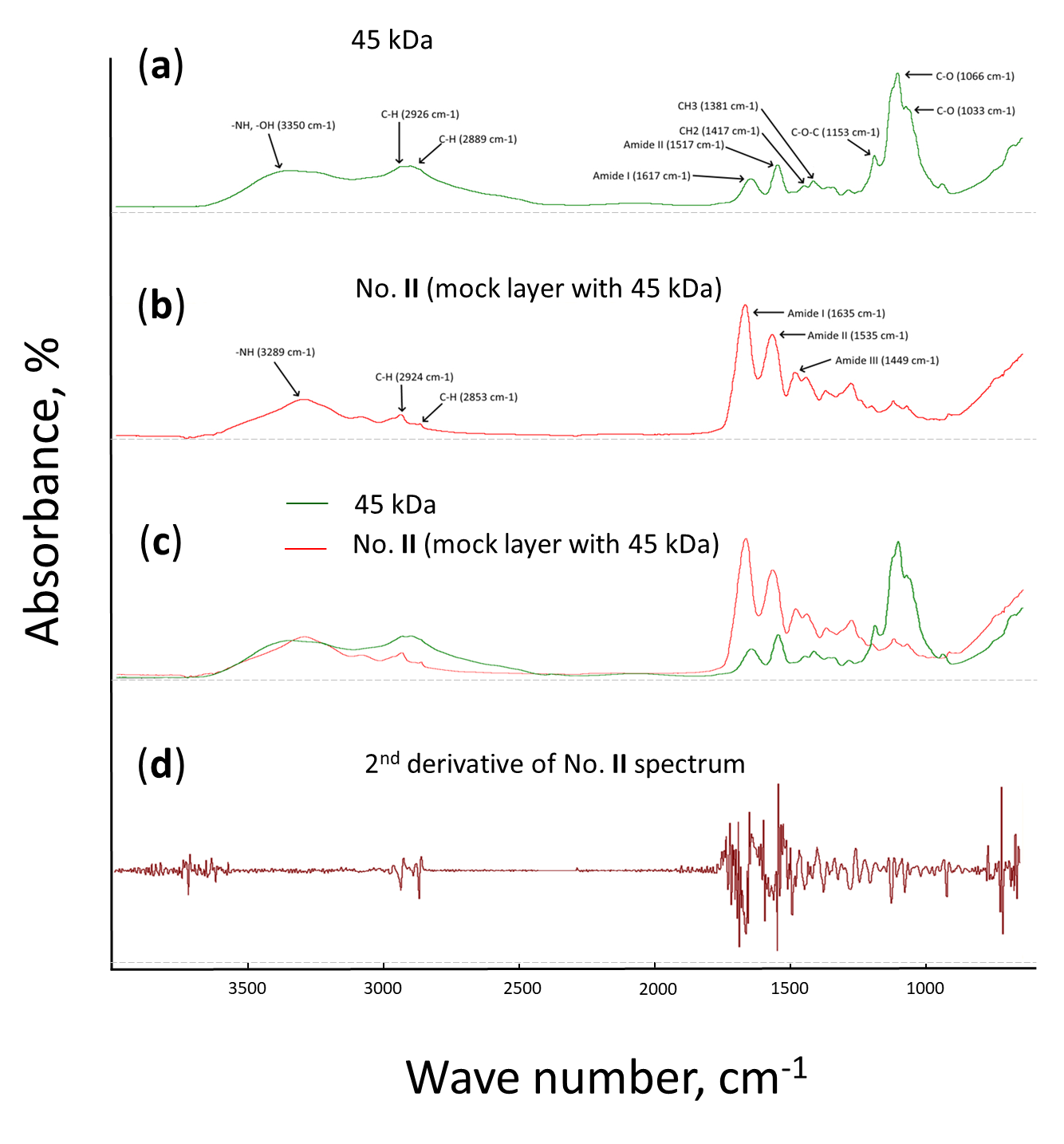

Supplement: Supplementary file 1 [file materials-15-07773-s001.zip › Figure S2.tif]

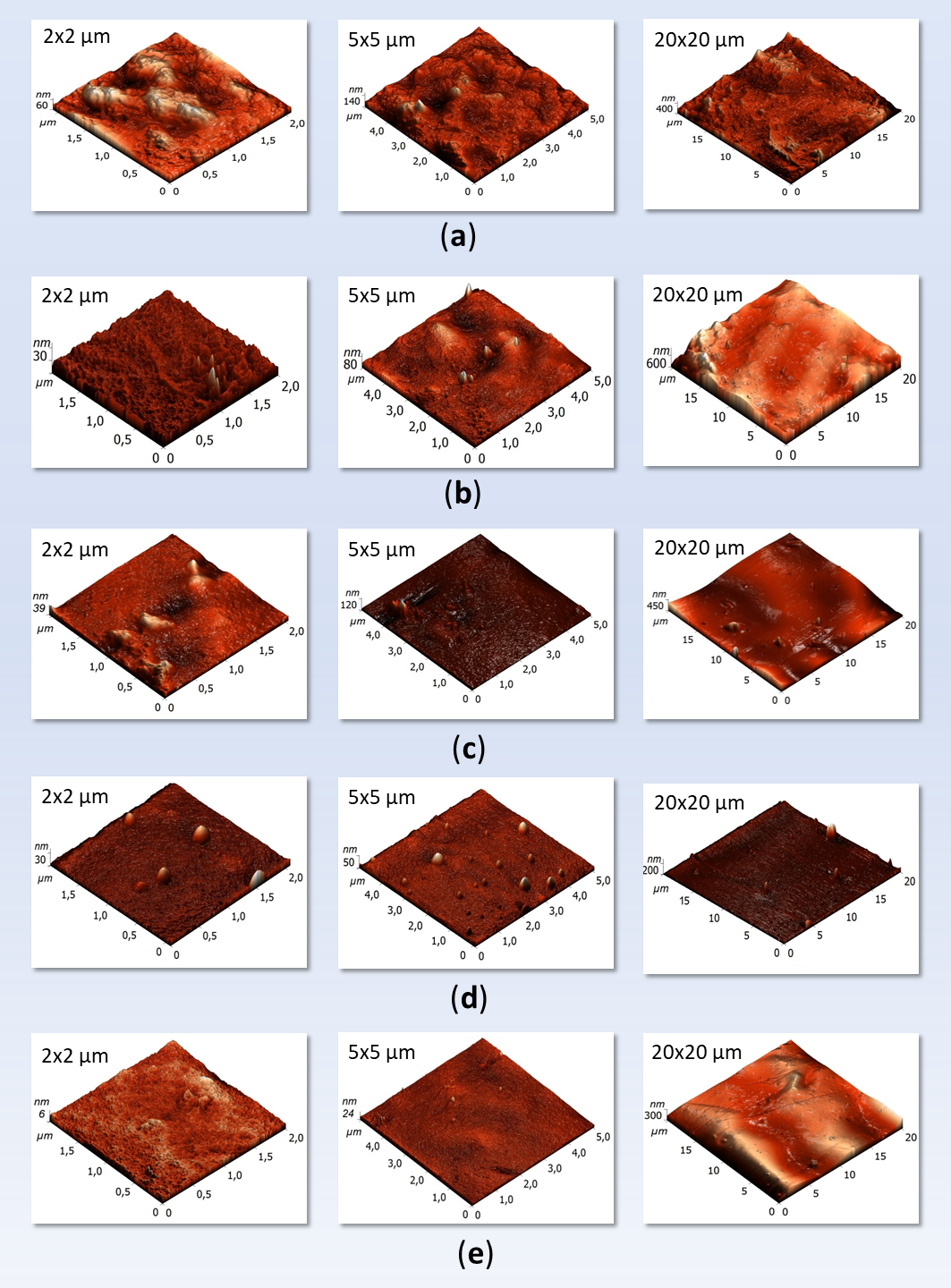

Supplement: Supplementary file 1 [file materials-15-07773-s001.zip › Figure S3.tif]

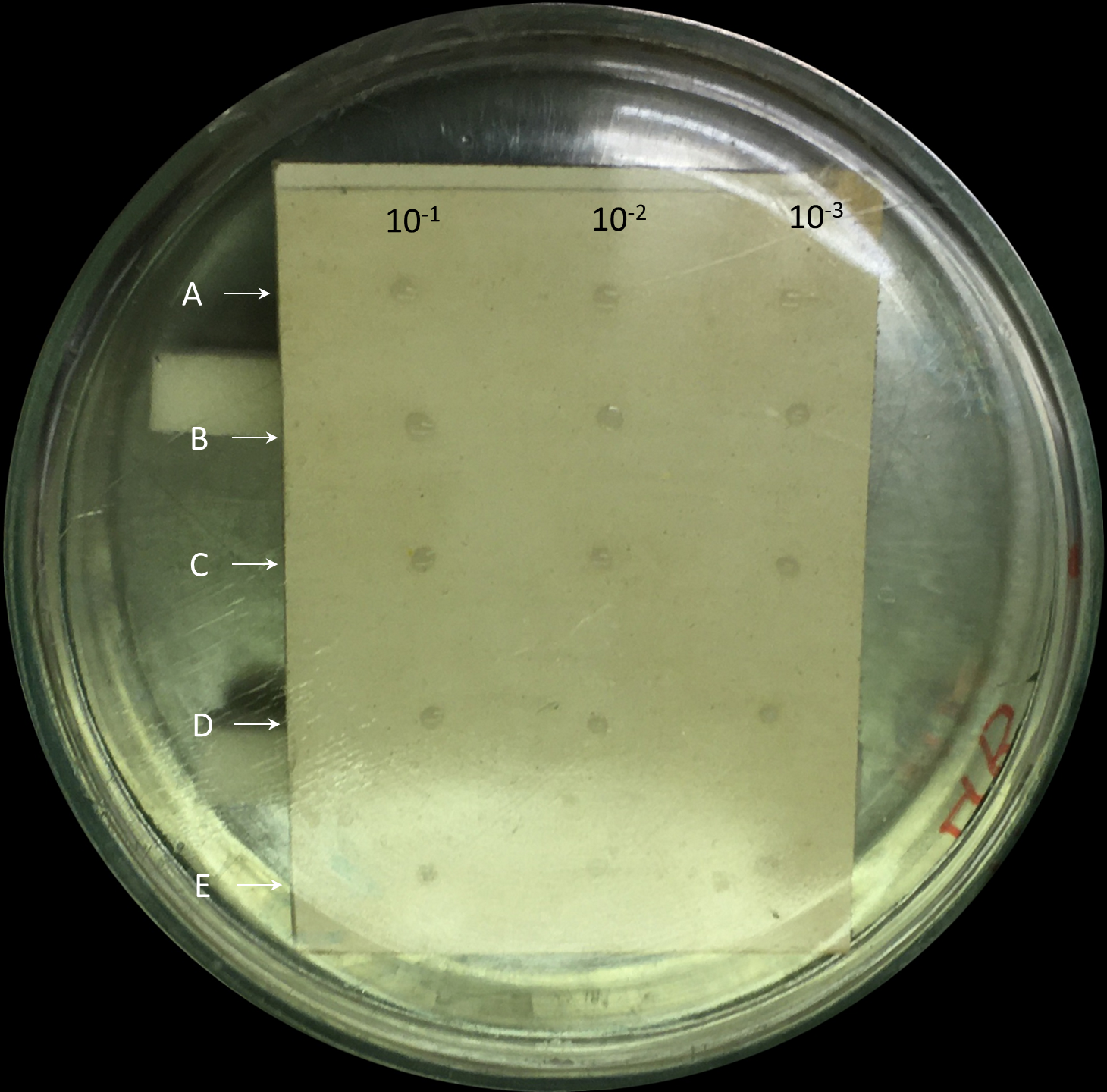

Supplement: Supplementary file 1 [file materials-15-07773-s001.zip › Figure S4.tif]

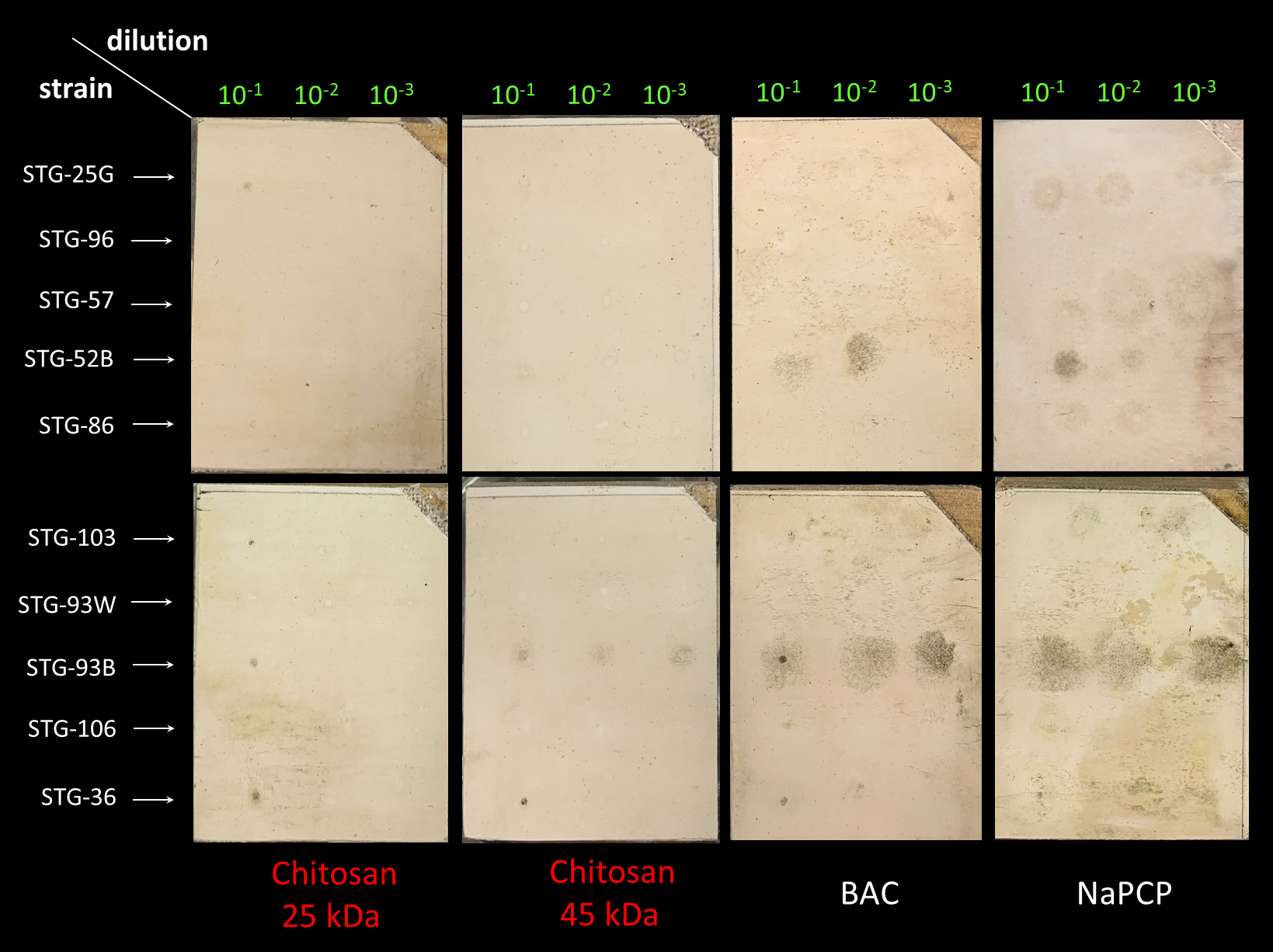

Supplement: Supplementary file 1 [file materials-15-07773-s001.zip › Figure S5.tif]
